# How to design and evaluate interventions to improve outcomes for patients with multimorbidity

**DOI:** 10.15256/joc.2013.3.21

**Published:** 2013-10-08

**Authors:** Susan M. Smith, Elizabeth A. Bayliss, Stewart W. Mercer, Jane Gunn, Mogens Vestergaard, Sally Wyke, Chris Salisbury, Martin Fortin

**Affiliations:** ^1^HRB Centre for Primary Care Research, Department of General Practice, RCSI Medical School, Dublin, Ireland; ^2^Kaiser Permanent Colorado and University of Colorado, Denver, Colorado, CO, USA; ^3^University of Glasgow, Glasgow, UK; ^4^University of Melbourne, Melbourne, VIC, Australia; ^5^Aarhus University, Aarhus, Denmark; ^6^University of Glasgow, Glasgow, UK; ^7^University of Bristol, Bristol, UK; ^8^Department of Family Medicine, University of Sherbrooke, Sherbrooke, Québec, Canada

**Keywords:** Multimorbidity, comorbidity, interventions, family practice, effectiveness, complex evaluations

## Abstract

Multimorbidity is a major challenge for patients and healthcare providers. The limited evidence of the effectiveness of interventions for people with multimorbidity means that there is a need for much more research and trials of potential interventions. Here we present a consensus view from a group of international researchers working to improve care for people with multimorbidity to guide future studies of interventions. We suggest that there is a need for careful consideration of whom to include, how to target interventions that address specific problems and that do not add to treatment burden, and selecting outcomes that matter both to patients and the healthcare system. Innovative design of these interventions will be necessary as many will be introduced in service settings and it will be important to ensure methodological rigour, relevance to service delivery, and generalizability across healthcare systems.

Journal of Comorbidity 2013;3:10–17

## Introduction

Multimorbidity, sometimes referred to as multiple chronic conditions, is defined as the co-existence of two or more chronic conditions in the same individual and includes both physical and mental health conditions [1]. Multimorbidity carries a substantial burden, not only for individuals, as it is associated with lower quality of life and lower levels of functioning, but also for healthcare delivery in terms of high utilization rates and complex care needs [2]. Prevalence increases with age, but in absolute terms there are more middle-aged people living with multimorbidity [3, 4]. Multimorbidity is also strongly associated with socioeconomic deprivation – with those in the most deprived groups developing multimorbidity approximately 10 years earlier than those in the most affluent groups [3]. Inter-related issues that add to the challenges of multimorbidity include: polypharmacy and adverse drug events, multifactorial symptom burden, treatment burden (as conditions and related treatments escalate), and the added complexity of combined physical and mental illness.

Despite the high prevalence of multimorbidity, evidence-based medicine and clinical practice guidelines are largely informed by trials focusing on single conditions and excluding patients with multimorbidity [5]. There is limited evidence of effectiveness of interventions, and a recent Cochrane review identified only 10 trials in this area [6]. Thus, there is a clear need to focus on intervention development and evaluation to address the challenges of multimorbidity [7]. Our experience as an international group actively engaged in multimorbidity research suggests that, although the Medical Research Council Framework for the Design and Evaluation of Complex Interventions to improve health outcomes [8] is both relevant and useful to guide research on interventions for multimorbidity, there are specific methodological issues relating to multimorbidity that need to be considered and addressed.

**Figure 1:**
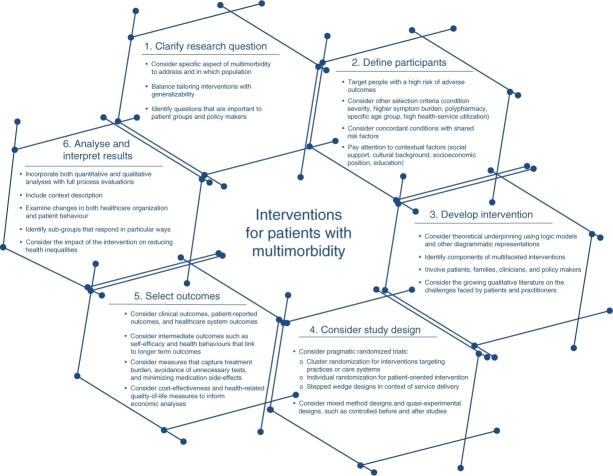
Interventions for patients with multimorbidity. Although outlined in sequence, these interventions need to be considered in an iterative fashion as each issue informs previous and subsequent decision-making.

This paper aims to guide researchers developing and evaluating interventions for patients with multimorbidity, and is based on a forum held at the North American Primary Care Research Group meeting in 2012. While the methodological issues are outlined in sequence, they need to be considered in an iterative fashion as each issue informs previous and subsequent decision-making, as illustrated by Figure 1.

## Clarify specific research question

Given the diversity of the population with multimorbidity, it is important to formulate a clear research question. Questions must focus on generalizable processes, while at the same time retaining the ability to assess the effectiveness of an intervention in specific subpopulations. The traditional Population, Intervention, Comparison, Outcome (PICO) format may require revisions. It may be best to initially focus on designing interventions that address specific outcomes, and subsequently identify subpopulations that are at risk for the outcome and are suited for the proposed intervention. For example, “How effective are pharmacist-led medicines reviews at reducing potentially inappropriate prescribing in patients with multimorbidity and polypharmacy?” Working closely with patient groups and policy makers to identify relevant outcomes may also help guide the development of appropriate questions.

## Define participants

The key issue when considering participant selection is to ensure external validity, while at the same time demonstrating effectiveness – often a challenge within this heterogeneous group [9]. There has been much discussion on the challenges of defining multimorbidity, which can range from disease counts, with or without restricted condition lists, to more sophisticated weighted scores incorporating symptom severity [10]. The most inclusive and simplest approach is to consider all individuals with two or more chronic conditions; though it is also necessary to clarify what is meant by a “chronic condition” [10]. However, this approach will include those who may not be in need of intervention, and others who may not be amenable to particular approaches. It may, therefore, be preferable to target people at higher risk of adverse outcomes who will be most likely to benefit from interventions. People with four or more conditions have poorer outcomes and escalating health-service utilization [11, 12]; so selecting participants with higher numbers of conditions may be more appropriate. Depending on the outcomes of interest, other selection criteria might include condition severity, symptom burden, polypharmacy, specific age groups, and high health-service utilization, including recent hospitalization.

Some interventions may lend themselves to populations having concordant conditions with shared risk factors. For example, an intervention to promote physical activity could benefit individuals with coexisting obesity, depression, and vascular risk factors. Interventions targeting patients with combined physical and mental health problems must also account for complex social factors, as this combination is most commonly associated with socioeconomic deprivation. Examples of other vulnerable subpopulations include migrants, those with cognitive impairment or literacy problems, and those with more complex and symptomatic conditions.

Target populations and inclusion and exclusion criteria must be carefully described to address the issue of generalizability and external validity. We also recommend paying particular attention to contextual factors that may affect generalizability by including a complete list of diagnoses of participants (with associated severity) as well as other factors, such as social support, cultural background and socioeconomic position.

## Developing the intervention: theoretical underpinning and potential components

Most interventions addressing multimorbidity are likely to be multifaceted and it is very important to have a conceptual understanding of how different intervention components are likely to affect outcomes. Determining causal pathways will necessarily be an iterative process, which can be aided by logic models and other diagrammatic representations. In this regard, interventions targeted at people with multimorbidity are no different to those targeted at other groups, and in a multifaceted intervention, there may be more than one theory or evidence base to draw on. For example, for interventions directed at individuals with multimorbidity, identifying specific techniques that support behavioural changes are particularly important [13]. However, for interventions addressing polypharmacy, the theory and evidence underpinning medicines management need to be considered.

The recent Cochrane review of interventions to improve outcomes for patients with multimorbidity adapted the Cochrane Effective Practice and Organisation of Care (EPOC) Group Taxonomy for interventions [14], which can also be used to identify components of multifaceted interventions, and facilitates consideration of the theoretical underpinning and evidence base for each component. The taxonomy is outlined in Table 1 with examples of potential interventions for patients with multimorbidity.

Participatory intervention development with explicit involvement of patients, families and carers, and clinical care providers or policy makers, consistent with the strong movement towards patient-centred outcomes research [15], may also be appropriate. As with identifying a research question, there is a need to balance tailoring interventions to the needs of individuals who have multimorbidity with delivering standardized interventions that are easier to coordinate, monitor, and evaluate. Intervention development may also be informed by the growing qualitative literature on multimorbidity that considers the patient and provider perspectives [16–18]. The combination of theory and evidence with practical experience, gained through participatory research, is likely to be powerful in developing interventions that operate at different levels. For example, recent research in areas of high deprivation in Scotland has taken a “co-production” approach: a complex primary care-based intervention to help people “live well with multimorbidity” has involved patients, healthcare professionals, voluntary organizations, and academics in the development, refinement, and optimization of the intervention [17].

## Consider study design

The most likely study designs in this context are pragmatic randomized trials. For interventions targeted at practices or care systems, cluster randomization may be ideal; however, this may require prohibitively large sample sizes. Study designs depend on the outcomes of interest, whether the outcome is measured at the level of the patient or the practice, and the expected effect size of the intervention. Interventions with large effect sizes may be feasible in terms of sample size, but important population-level interventions, such as emergency admissions, may have relatively small effect sizes [19]. The use of intermediate process outcomes, known to be linked to outcomes, such as admissions and Emergency Department visits, may help get around the need for large sample sizes. Individual randomization is feasible for some of the more patient-oriented interventions, such as self-management support programmes or specifically designed occupational health or physiotherapy interventions that also lend themselves to the use of waiting-list controls.

More imaginative approaches to study design include stepped wedge designs that have the advantage of allowing incorporation of service delivery in a phased way and which can also increase power. This design can be adopted in the context where phased service development is planned in an area and where it would be unacceptable to randomize participants to waiting lists or usual care within the same centre. This may be particularly useful for patients with more complex multimorbidity when randomization is not feasible or practitioners do not feel comfortable randomizing such patients to usual care or waiting-list controls. For example, a study in Australia using Mental Health Experience Co-Design (MH ECO) methodology (see http://mheco.org.au/) involves eight community health centres that are being brought into the intervention in random order at intervals over 4 years. This design is useful when the number of clusters is very low and the research team has no control over their number. For best results, the stepped wedge design requires that interventions are likely to result in rapid and large changes in outcomes. However, this design is no panacea and will usually require more outcome measurements and more time than cluster randomized trials [20]. Mixed method designs incorporating both quantitative and qualitative methods are likely to be of particular importance given the complexity of populations and interventions. Quasi-experimental designs, such as controlled before and after studies, may also be considered for pragmatic reasons as they may be more acceptable for patients with multimorbidity in service-delivery settings. The key issue when choosing any design is that it is robust enough to contribute to the existing evidence and be incorporated into systematic reviews [21].

## Selecting outcomes

The key aspect of outcome selection is matching outcomes to the proposed mechanisms of action for each component of the intervention. We have found it helpful to group outcomes into clinical outcomes, patient outcomes or patient-reported outcome measures (PROMs), and healthcare system outcomes. These are described in more detail here with examples given in Table 2. Clinical outcomes are challenging in a population with a variety of conditions, and innovative approaches are required to assess physical health outcomes. A good example of a potentially useful approach for broadly designed interventions has been developed by Reeves *et al.,* who combined multiple indicators of clinical quality across a range of 23 acute and chronic conditions, and measured the proportion of disease targets, which were met for each condition [22].

Intermediate outcomes (sometimes thought of as mediating variables, such as self-efficacy or health behaviours) that are associated with “hard outcomes”, such as hospital admission or mortality, are particularly useful, as many “hard outcomes” may be difficult to assess directly due to long follow-up times or difficulty with large-scale data collection. These associations can be illustrated in a logic model to demonstrate the relationship between more proximal and distal outcomes.

Disease-specific outcomes may not be appropriate as primary outcomes in patients with multimorbidity because (i) there will be multiple combinations of individual conditions and (ii) they may have less to gain from ideal control of each individual disease. The guidance for individual diseases may not be applicable to these complex patients because the underlying research excluded individuals with comorbidities, and maximizing treatment may not reflect their own priorities, especially if it leads to polypharmacy and extensive investigation. A consequence of maximizing care for individual diseases could be excessive treatment burden. A validated treatment burden measure is currently being developed which may be particularly useful for the population with multimorbidity [23]. Other outcomes might include avoidance of unnecessary tests and minimizing medication side effects. In the setting of multimorbidity, an intervention that aims to be responsive to people’s own priorities and reduces over-treatment may conceivably lead to reduced performance on conventional measures of disease control (e.g. tight control of blood pressure) while still successfully achieving its aims. However, the focus on the patients’ agenda must be balanced by clinical knowledge and, in some cases, a focus on individual condition management may remain important. For example, there is sufficient evidence to support optimal medical management of congestive cardiac failure, both in reducing mortality and in reducing admissions and troublesome physical symptoms, such as breathlessness. However, an agenda entirely driven by patient priorities may not recognize this, highlighting the importance of shared decision-making and clinical expertise. Generic outcomes that focus on quality of life and functional capacity should also improve with optimal medical management, where this is evidence-based and appropriate.

Finally, health systems will be particularly interested in identifying cost-effective processes of care that minimize unintended consequences for the population with multimorbidity. The incorporation of health-related quality-of-life outcomes will enable economic analyses with a broader perspective in terms of additional quality-adjusted life years gained.

## Analysis and interpretation of results

Analysis of results will need to follow recommended guidelines based on trial design (see www.equator-network.org). Careful process evaluations incorporating both quantitative methods (e.g. how many people received the intervention as intended) and qualitative methods (e.g. how the intervention is implemented, what worked, and what did not work) will be needed to document how interventions are operationalized and to allow replication or potential adaptations for other settings [24]. This should include description of the context, i.e. the patient population, the community, and the healthcare setting, as a means of understanding whether and how an intervention will generalize to other settings. A formal process evaluation should also assess treatment burden as a result of participation in the intervention itself. Many interventions will be designed to work at two levels: (i) changes in healthcare organization and/or clinician behaviour, which are intended to lead to (ii) changes in patient behaviour. It is important to examine both levels in a process evaluation. An intervention may fail because it is not fully implemented by the clinicians, or because it is fully implemented, but is not effective at changing patient behaviour.

Disease type and severity may also influence outcomes; therefore, accurate measurement of morbidity (including self-reported morbidity burden or symptom severity) becomes important in interpreting results. With multimorbidity, it is important to try to identify subgroups who respond in particular ways, and to examine the likely impact of the intervention on reducing inequalities in health, though such analyses may require boosting of sample sizes. Investigators undertaking cluster randomized trials should also publish the observed intra-cluster correlation coefficients for key outcomes, to inform the sample size calculations for future studies.

## Conclusion

Multimorbidity is increasingly important and we need cost-effective interventions to improve outcomes in this group of patients in all healthcare systems. People with multimorbidity are, by definition, a heterogeneous group, and different interventions may be required for different subgroups, depending on condition severity, condition combinations, social circumstances, or age. Multimorbidity intervention research raises substantial ethical issues that need to be tackled – the obvious one being, “who decides what is a good outcome?” Involvement of patients, family members, clinicians, and policy makers in developing research questions and interventions would help address these issues. This reinforces the importance of good qualitative research, both to develop and evaluate interventions and to contribute to the debate. There is a need to clearly report participant characteristics, intervention components and delivery, and results in a way that enables comparison across trials, and to ensure that future trials can be incorporated into updated systematic reviews. Multimorbidity research will also illustrate, and perhaps magnify, tensions present in patient-centred care delivery, which involve prioritization and shared decision-making and making choices in the face of multiple potential outcomes.

## Figures and Tables

**Table 1:** Taxonomy of interventions.

Intervention type	Example and how it might work
Professional interventions	Intervention designed to change the behaviour of clinicians; for example, by altering professionals awareness of multimorbidity or providing training or education designed to equip clinicians with specific skills in managing multimorbidity, including supporting patients, to prioritize their concerns or enhance shared decision-making
Financial interventions	Financial incentives to providers; for example, incentivizing health-service delivery and providing resources to extend consultation length for patients with multimorbidity
Organizational interventions	Organizational changes; for example, any changes to care delivery such as case management or integrating the work of different healthcare workers, such as a pharmacists and general practitioners. Such interventions may work by re-orienting care delivery to match the specific needs of patients with multimorbidity, including care coordination, medicines management, or specific physiotherapy or occupational therapy interventions to address needs relating to physical and social functioning. These may also include technological or information technology interventions designed to enhance care coordination and communication
Patient-oriented interventions	This would include any intervention directed primarily at patients; for example, to help make changes to improve well-being, or focus on areas of lifestyle important to them (such as losing weight or increasing physical activity). Patient education or support for self-management, which might work by improving self-management, thus enabling patients to manage their conditions more effectively and to seek health care more appropriately. Interventions to improve shared decision-making and prioritization of problems
Regulatory interventions	Changes to local or national regulations designed to alter care delivery; for example, inclusion of annual medication reviews in contracts with care providers

**Table 2:** Potential outcomes in multimorbidity research.

Domain	Example of measure	Comment
*Clinical*		
Disease-specific measures		Only relevant for comorbidity studies where interventions directed at all included comorbid conditions
Clinical quality indicators		Promising approach assessing proportions of indicators met across conditions
Risk factors	BP, lipids	May be more relevant for certain condition combinations
Body weight	Weight/BMI, waist–hip ratios	More relevant across certain conditions
Frailty/physical fitness		The measures frequently require clinical assessment, physical testing or at a minimum patient self-report
*Patient-reported outcome measures*		
Psychological	Self-efficacy	Need clear link with theoretical underpinning of intervention
	HRQoL	
	Well-being	
	Measures of anxiety and depression	
Behaviour and daily functioning	Physical functioning	
	Activities of daily living	
	Self-management behaviours	
	Health behaviours	
	Number of days out of role	
	Smoking	May be more relevant in longer-term studies when trying to prevent further decline
Social	Social inclusion and participation	
	Social support	
	Patient engagement and empowerment	
Treatment burden		
Shared decision-making		
Goal setting		
Satisfaction with care provision		
*Healthcare system*		
Health-service utilization	Provider visits	Can be hard to determine what is appropriate change, depending on baseline provision
	Admissions	Determine which admissions are sensitive to changes in ambulatory-care delivery
Process of care	Risk-factor recording	May be more relevant for specific condition combinations
	Annual reviews	Depends on goals of care
Accessibility of services		
Safety indicators	Adverse drug events	Challenging to measure due to inconsistent documentation and need for expert chart review
*Healthcare provider measures*		
Satisfaction with care delivery		
“Burn-out”		
Confidence and competence in deliver care	Self-efficacy	Confidence in ability to deliver care
	Knowledge	May be relevant for medicines management
	Skill	Ability to deliver behavioural change based interventions
*Cost outcomes*		
Costs of care		
Indirect costs		
*Other outcomes*		
Carer burden		Including outcomes for children of patients with multimorbidity
Alignment of treatment goals between patients and providers		

BMI, body mass index; BP, blood pressure; HRQoL, health-related quality of life.

## References

[1] Fortin M, Soubhi H, Hudon C, Bayliss EA, van den Akker M (2007). Multimorbidity’s many challenges. Br Med J.

[2] International Research Community on Multimorbidity Available from: http://www.usherbrooke.ca/crmcspl/en/international-research-community-on-multimorbidity/ [Last accessed Apr 12, 2013].

[3] Barnett K, Mercer SW, Norbury M, Watt G, Wyke S, Guthrie B (2012). The epidemiology of multimorbidity in a large cross-sectional dataset: implications for health care, research and medical education. Lancet.

[4] Taylor AW, Price K, Gill TK, Adams R, Pilkington R, Carrangis N (2010). Multimorbidity - not just an older person’s issue. Results from an Australian biomedical study. BMC Public Health.

[5] Fortin M, Dionne J, Pinho G, Gignac J, Almirall J, Lapointe L (2006). Randomized clinical trials: Do they have external validity for patients with multiple comorbidities?. Ann Fam Med.

[6] Smith SM, Soubhi H, Fortin M, Hudon C, O’Dowd T (2012). Managing patients with multimorbidity: systematic review of interventions in primary care and community settings. Br Med J.

[7] Salisbury C (2013). Multimorbidity: time for action rather than words. Br J Gen Pract.

[8] Craig P, Dieppe P, Macintyre S, Michie S, Nazareth I, Petticrew M (2008). Developing and evaluating complex interventions: the new Medical Research Council guidance. Br Med J.

[9] Mercer SW, Smith SM, Wyke S, O’Dowd T, Watt GC (2009). Multimorbidity in primary care: developing the research agenda. Fam Pract.

[10] Valderas JM, Starfield B, Sibbald B, Salisbury C, Roland M (2009). Defining comorbidity: implications for understanding health and health services. Ann Fam Med.

[11] Centers for Medicare & Medicaid Services (2011). Chronic Conditions among Medicare beneficiaries. Chart Book.

[12] Glynn LG, Valderas JM, Healy P, Burke E, Newell J, Gillespie P (2011). The prevalence of multimorbidity in primary care and its effect on health care utilization and cost. Fam Pract.

[13] Dombrowski SU, Sniehotta FF, Avenell A, Johnston M, MacLennan G, Arajo-Soares V (2012). Identifying active ingredients in complex behavioural interventions for obese adults with obesity-related co-morbidities or additional risk factors for co-morbidities: a systematic review. Health Psychol Rev.

[14] Smith SM, Soubhi H, Fortin M, Hudon C, O’Dowd T (2012). Interventions for improving outcomes in patients with multimorbidity in primary care and community settings. Cochrane Database Syst Rev.

[15] Concannon TW, Meissner P, Grunbaum JA, McElwee N, Guise JM, Santa J (2012). A new taxonomy for stakeholder engagement in patient-centered outcomes research. J Gen Intern Med.

[16] Noel PH, Parchman ML, Williams JW, Cornell JE, Shuko L, Zeber JE (2007). The challenges of multimorbidity from the patient perspective. J Gen Intern Med.

[17] O’Brien R, Wyke S, Guthrie B, Watt G, Mercer S (2011). An ‘endless struggle’: a qualitative study of general practitioners’ and practice nurses’ experiences of managing multimorbidity in socio-economically deprived areas of Scotland. Chronic Illn.

[18] Smith SM, O’Kelly S, O’Dowd T (2010). GPs’ and pharmacists’ experiences of managing multimorbidity: a ‘Pandora’s box’. Br J Gen Pract.

[19] Roland M, Abel G (2012). Reducing emergency admissions: are we on the right track?. Br Med J.

[20] Kotz D, Spigt M, Arts IC, Crutzen R, Viechtbauer W (2012). Use of the stepped wedge design cannot be recommended: a critical appraisal and comparison with the classic cluster randomized controlled trial design. J Clin Epidemiol.

[21] Antes G, Clarke M (2012). Knowledge as a key resource for health challenges. Lancet.

[22] Reeves D, Campbell SM, Adams J, Shekelle PG, Kontopantelis E, Roland MO (2007). Combining multiple indicators of clinical quality: an evaluation of different analytic approaches. Med Care.

[23] Tran VT, Montori VM, Eton DT, Baruch D, Falissard B, Ravaud P (2012). Development and description of measurement properties of an instrument to assess treatment burden among patients with multiple chronic conditions. BMC Med.

[24] Grant A, Treweek S, Dreischulte T, Foy R, Guthrie B (2013). Process evaluations for cluster-randomised trials of complex interventions: a proposed framework for design and reporting. Trials.

